# In Commemoration of Dr. Bijan Sadrizadeh, a Prominent Physician and Expert in the Field of Public Health in Iran and Around the World

**DOI:** 10.34172/aim.2023.09

**Published:** 2023-01-01

**Authors:** Fatemeh Bardestani, Seyed Alireza Marandi, Reza Malekzadeh, Abolhassan Nadim, Hossein Malekafzali, Kamran Bagheri Lankarani, Mohsen Bavandi, Alireza Mesdaghinia, Mohammad Mehdi Gouya, Roya Sadrizadeh, Ehsan Mostafavi

**Affiliations:** ^1^Research Centre for Emerging and Reemerging Infectious Diseases, Pasteur Institute of Iran, Tehran, Iran; ^2^Academy of Medical Sciences, Tehran, Iran; ^3^Digestive Disease Research Center, Shahriari Hospital, Tehran University of Medical Sciences, Tehran, Iran; ^4^School of Public Health, Tehran University of Medical Sciences, Tehran, Iran; ^5^Health Policy Research Center, Institute of Health, Shiraz University of Medical Sciences, Shiraz, Iran; ^6^School of Medicine, Iran University of Medical Sciences, Teheran, Iran; ^7^Ridgeway, Ontario, Canada, LOS 1N0

**Keywords:** History of medicine, Polio, Public health

## Abstract

In line with the commemoration of the scientists who played a significant role in advancing knowledge and providing services to the country, it is imperative to publish their biographies so that their lives and achievements are recorded in the history of the country and serve as an example for future generations. Dr. Bijan Sadrizadeh, a physician and a public health specialist, undertook many valuable activities, particularly in the field of public health in Iran and the world during more than 60 years of great services, including the promotion of public health in the Islamic Republic of Iran, the development of I.R. Iran’s international collaborations in the field of public health, and the development of research programs in the field of neglected tropical diseases and the eradication of polio in the world. He served the country in many high-level executive capacities, including three periods as deputy Minister of Health. In addition to several years of full-time employment in the World Health Organization (WHO), Dr. Sadrizadeh also served on the WHO Executive Board and was a member of numerous scientific and advisory committees. In reviewing his life, great determination, devotion, believing in primary health care and universal health coverage and a deep sense of responsibility are visible and can be an inspiration and a model for all.

## Introduction

 The late Dr. Bijan Sadrizadeh was born on February 20, 1938, in the village of “Irancheh”, in Farrokhshahr, the district of Shahrekord in Chaharmahal and Bakhtiari province, Iran. He received his medical degree from Isfahan University of Medical Sciences, and obtained a Master of Public Health (MPH) from the Institute of Tropical Medicine in Belgium, and a speciality in Infectious Diseases and Tropical Medicine from Tehran University of Medical Sciences. He started his medical profession in a remote village. Gradually, with hard work, discipline and competence, he worked his way up to becoming deputy to the Minister of Health and becoming a prominent international public health person. Dr. Sadrizadeh had a passion for strengthening Iran’s primary healthcare system and played a significant role in developing one of the best healthcare networks in the world for his country.

**Figure 1:**
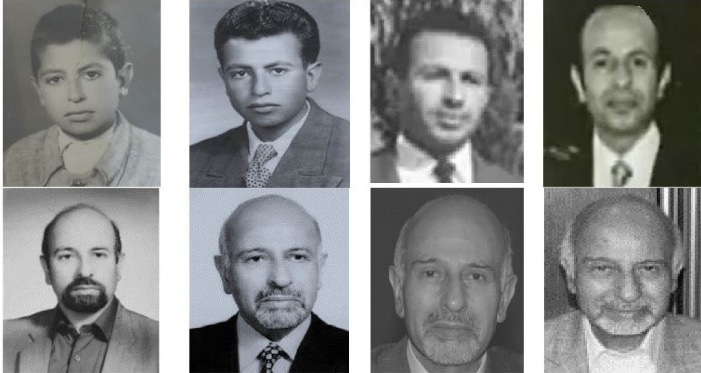
Dr. Bijan Sadrizadeh Over Time

 In addition to his eight years of full-time service in the World Health Organization (WHO), Dr. Sadrizadeh also served as a member of the WHO’s Executive Board Committee, chairman of the Special Program for Research and Training in Tropical Diseases, a member of the Health Research Advisory Group, and a member of the Advisory Group for the Neglected Tropical Diseases. He was also a member of the WHO Technical Advisory Group on Influenza Vaccine Production, a member of the Eastern Mediterranean and also the African Regional Certification Commission (RCC) for polio eradication, and a member of the Technical Advisory Group on Polio Eradication in Afghanistan and Pakistan (Figure 1).

 He passed away on July 24, 2022, at the age of 85. In this paper, we briefly review some aspects of Dr. Sadrizadeh’s life and professional function.

## Education and Scientific Degrees

 Bijan Sadrizadeh spent his primary school years in the villages close to the village of “Irancheh” in the province of Chaharmahal and Bakhtiyari. After completing six years of elementary school, he attended high school in the cities of Borujen and Isfahan. After receiving his high school diploma, Sadrizadeh was admitted to Isfahan Higher School of Health Education in 1957 and completed the first four-year course in general medicine. After serving in the rural areas for a few years, he continued his medical education for another four years and received his degree as a medical doctor from Isfahan University of Medicine Sciences in 1969.

 He received a Master in Public Health from the Institute of Tropical Medicine in Belgium, in 1978, followed by a supplementary course at the University of Oslo, Norway. In 1989, Dr. Sadrizadeh obtained a speciality in infectious diseases and tropical medicine from Tehran University of Medical Sciences. During his studies, he benefited from the presence of professors such as Dr. Abu Torab Nafisi, Dr. Mohammad Riahi, Dr. Morteza Hakami, and Dr. Alireza Yalda.

 Between 1990 and 1992, he taught as an assistant professor at Shahid Beheshti University of Medical Sciences in Tehran. In 1992, he retired from his positions in the country and joined the WHO. Dr. Sadrizadeh served in various capacities during his 8-year tenure at the WHO. He returned to Iran in 2000 and served the health system of the Islamic Republic of Iran in various positions until the end of his life.

## Executive Activities

 Dr. Sadrizadeh spent more than 60 years of his fruitful life in scientific and managerial activities in the Islamic Republic of Iran and the WHO. In the following, we will review these activities briefly:

###  A- Executive activities in the Islamic Republic of Iran

**Figure 2:**
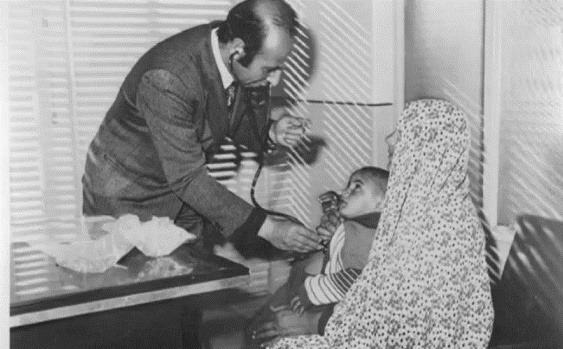
Dr. Bijan Sadrizadeh During His Medical Practice in Miami, Shahrood in 1970

 Dr. Sadrizadeh spent four years of his medical services, as an MBBS in Medicine, in the villages of Damghan (1961–1965), and three years as a Doctor of Medicine (MD) in Shahmirzad (1971–1974), both in the Semnan province. After completing his studies in general medicine, he joined the Health Corps for military service and served in Miami Shahrood in 1969 and 1970 (Figure 2).

 During his life, he held many executive responsibilities. His entry into the executive branch of health was in the Semnan province between 1974 and 1980 when he served as the Head of the Semnan Department of Communicable Diseases Control and Malaria Eradication of the Semnan province (1974–1978), the Head of the Public Health Network in the Semnan province (1979), and the Head of Health and Welfare Organization of the Semnan province (1980).

**Figure 3:**
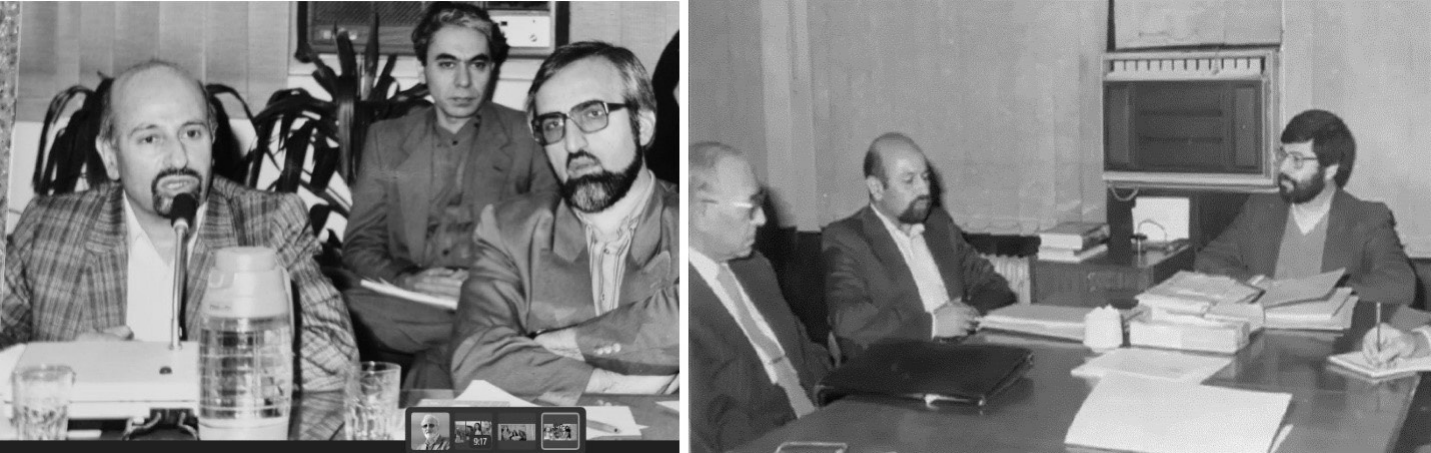
Dr. Bijan Sadrizadeh as Deputy Minister of Health with Dr. Seyed Alireza Marandi (1987, right), and Dr. Reza Malekzadeh (1991, left), Ministers of Health at the time

 Dr. Sadrizadeh was transferred to Tehran in September 1980 and served as the Director General of the Communicable Disease Control and Malaria Eradication Center of the Ministry of Health until 1982. Then, he served as Director General of the Family Health Office of the Ministry of Health (3 years), The director of the Headquarters for the Development of Primary Health Networks in the Country (1 year), Deputy Minister of Public Health (working with three ministers of Health and Medical Education: Dr. Seyed Alireza Marandi, Dr. Iraj Fazel and Dr. Reza Malekzadeh, for a total of 5 years) (Figure 3).

 Dr. Sadrizadeh played an important role in expanding primary health care and health centers in the country, particularly in more remote and disadvantaged parts of the country, ultimately improving the health situation of villagers during his tenure as deputy health minister.

 He increased the number of health centers and also the living facilities for physicians who resided and worked in the villages. He also worked on the development of birth centers run by midwives in rural areas, which played an important role in reducing mother and child mortality rates in the country. After the end of the 8-year imposed war on Iran, insufficient funding was the main obstacle in the way of improving and expanding the health networks in rural areas. Under his leadership, the problem of resource shortage was largely solved by the savings achieved by reducing the consumption of baby milk formula, and the assistance from the World Bank. The savings were re-located to the completion of the health network by adding 4340 health centers and building additional 350 rural health centers and 300 rural maternity wards in the country.

 He also had an instrumental role in the development of the National Diarrheal Disease Control Program (1985), the National HIV/AIDS Program (1986), the National Immunization Development Program (1987), and the Acute Respiratory Infection Control Program (1988).

 In 2000, he completed his 8 years of service at the WHO, returned to his country and continued to serve as Advisor on Health and International Affairs to four ministers of health (Dr. Mohammad Farhadi, Dr. Masoud Pezeshkian, Dr. Kamran Bagheri Lankarani, and Dr. Marzieh Vahid Dastjardi) (2000–2009), president of the National Committee for the Confirmation of Polio Eradication in Iran (2002–2022), and member of the National Immunization Committee (2003–2022). He also served as a member of the Board of Trustees of Shahrekord University of Medical Sciences for twenty years (2001–2022), he was a member of the Board of Directors of Scientific Associations of Iranian Medical Group (2002–2022) and the President of Iranian Health Scientific Association (2000–2021).

 Another important and prominent period in Dr. Sadrizadeh’s service began when he joined the Iran Academy of Medical Sciences as a member of the Health and Nutrition Department in 2000, a position he held for 22 years until the end of his life. During this time, he served as Advisor to the President of the Academy of Medical Sciences on Health and International Affairs and Deputy Head of the Academy’s Department of Health and Nutrition (2009–2022).

###  B- Executive activities in the WHO

 In 1992, Dr. Sadrizadeh started his work at the WHO office in Sudan. In the first two years, he was the expert responsible for the development of medical personnel and health management. Later, due to his hard work and significant achievements, especially in combating infectious diseases, he was appointed as WHO’s Representative in Sudan for two more years to continue his effective activities.

 In 1996, Dr Sadrizadeh was transferred to WHO’s regional office for the Eastern Mediterranean Region in Egypt, and assumed high-level responsibilities in that organization, including the role of Regional Advisor for the Development of Health Management in the organization’s Eastern Mediterranean Region (1 year, Alexandria, Egypt) and Director for Communicable Diseases Control in the Eastern Mediterranean Region (3 years, Alexandria, Egypt). During this period, he was a member of the Medical Research Committee of the Eastern Mediterranean Region of the WHO (2 periods of 3 years), a member of the Joint Coordinating Board for Research and Training in Tropical Diseases (3 periods of 3 years), member of the Advisory Committee of the Global Immunization Program (1 year), Member of the Executive Council of the WHO (2 periods of three years), the deputy of the Executive Council of the WHO (1 year), and the deputy of the Joint Coordinating Board for Research and Training in Tropical Diseases (1 year).

**Figure 4:**
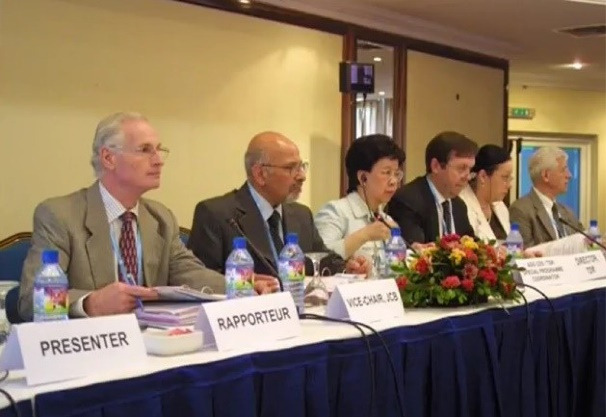
Dr. Bijan Sadrizadeh at one of the World Health Organization meetings as Vice President next to Dr. Margaret Chan, Secretary General of the World Health Organization in 2006

 After Dr. Sadrizadeh’s return to Iran in 2000, he continued his contribution to international public health by serving as Vice Chairman of the International Group for the Review and Revision of the International Health Regulations (IHR) (Geneva, 2004), Chairman of the Committee A of the 58th World Health Assembly (Geneva, 2005), Chairman of the Joint Coordinating Board for Research and Training in Tropical Diseases (2005 and 2006) (Figure 4), member of the Strategic Technical Advisory Committee on Neglected Tropical Diseases (2007–2009), and member of the WHO Work Program Advisory Group on Production of Pandemic Influenza Vaccine (2007–2009). From 2013 until his demise in 2022, he was a member of the WHO’s Commissions for the Eradication of Polio in the Eastern Mediterranean Region and the African Region, and a member of the WHO’s Advisory Group for the Eradication of Polio in Afghanistan and Pakistan. The eradication of polio in Africa in 2020 was certified by a 15-member committee consisting of Dr. Bijan Sadrizadeh.^1^

 During his service, Dr. Sadrizadeh participated in more than 50 scientific meetings and training courses as a consultant to the WHO.

## Scientific Activities

 He participated in the writing of the book Epidemiology of HIV/AIDS,^2^ one of the chapters of the comprehensive book on public health^3^ and in the translation of the book Broad Programming as Part of the Managerial Process for National Health Development^4^ and Diarrhea Disease Control Program.^5^ He also served on the editorial board of the book Infectious Disease Control, published by the American Public Health Association.

 He was a member of the editorial board of World Health Journal (2000–2022), a member of the editorial board of Iran Health Journal (2000–2022), and a member of the editorial board of the Journal of School of Public Health and Institute of Public Health Research (2002–2022).

 He wrote numerous scientific papers and reports in the field of polio,^6-12^ HIV/AIDS,^13-15^ immunization,^16,17^ malaria,^18,19^ measles,^20^ and the health care system of the Islamic Republic of Iran.^16,21-23^ Dr. Sadrizadeh’s papers have also been published in other public health fields.^24-27^

## Awards and Honors

**Figure 5:**
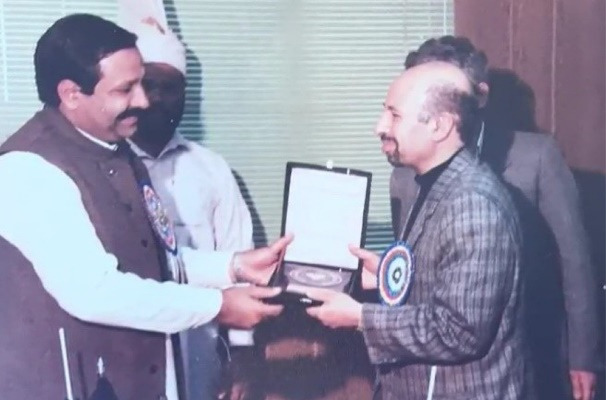
Dr. Bijan Sadrizadeh receives a plaque of honor in 2000 for his services to the Eastern Mediterranean region

 During his service, Dr. Bijan Sadrizadeh was honored numerous times at the national and international levels. Major awards include several plaques of honor from the WHO including Plaque of Honor for Effective Service in the Eastern Mediterranean Region (2000) (Figure 5), Plaque of Honor for Effective Collaboration with the Global Program of Education and Research in Tropical Diseases, WHO’s Golden Hammer Award (2005), Special award in the Anniversary of the Establishment of the School of Public Health of Tehran University of Medical Sciences (2016), the Superior Scientist Medal of Honor awarded by Professor Yalda Foundation (2016), the Health Award of the Academy of Medical Sciences (2016), a Presidential Certificate of Recognition as an elite of the country’s health sector, and many recognitions from the country’s Ministers of Health (in honor of his service to the country’s health development).

## Family

 Dr. Bijan Sadrizadeh was the second child of the family and had two brothers and two sisters. His father, the late Abdul Karim, was a farmer who could read and write well. His mother was a housewife and could read the Quran and Hafiz poems. In his memoirs, Dr. Sadrizadeh cites his mother’s reading of Hafez as one of the reasons for his interest in the poet. For him, trust in God and perseverance were the hallmarks of his life and the moral legacy of his mother.

**Figure 6:**
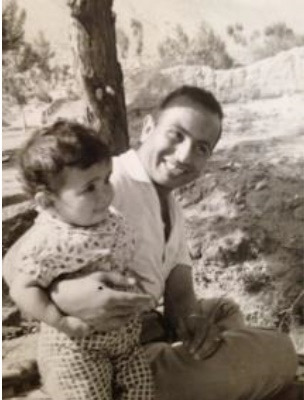
Dr. Bijan Sadrizadeh During His Medical Service Project in Rural Damghan in 1965 with His First Daughter, Roya

 He got married in 1963 and started his life in the villages around Semnan, where he worked as a practitioner. Dr. Sadrizadeh had four children named Roya, Babak, Mozhgan and Azadeh (Figure 6). His son passed away at the age of 31.

## Personality

 Dr. Sadrizadeh was known as a self-made man, hardworking, tireless, persistent, very disciplined, punctual, conscientious, caring, modest, religious, intelligent, contented, philanthropic and moral.

 He was a master in the field of public health and made great efforts to enhance public health in Iran and around the world. He took every opportunity to enhance Iran’s position and role in global health.

 He believed that the secret to his success in life was trust in God, being conscientious, perseverance, and following a step-by-step path to professional success starting from serving in remote villages. His main concern during his service in the Ministry of Health was to improve the health network throughout the country. In the truest sense of the word, he loved serving people, especially the country’s rural population.

 He learned and became proficient in English by self-study. He had beautiful handwriting and an excellent memory so he began to memorize the poems of Hafez, at the age of 70, and by the time of his death, he had memorized 333 poems or more than three-quarters of Hafez’s poems. Professor Bahauddin Khorramshahi in his book “*Hafez and Hafez Research*” named Dr. Sadrizadeh as one of the few people who knew the chief part of Hafez’s collection of poems by heart.

## Conclusion

 Dr. Bijan Sadrizadeh was a distinguished personality and a prominent name in the field of public health, who has left many legacies both at national and international levels. A review of the professional and personal life of this self-made and hardworking physician can provide a role model for current and future generations of this country.

 We will finish this article with verses from Hafez, which Dr. Sadrizadeh often read in his memoirs, advising people to be patient with problems:

 باغبان گر پنج روزي صحبت گل بايدش

 برجفاي خار هجران، صبر بلبل بايدش

 اي دل اندر بند زلفش از پريشاني منال

 مرغ زيرک چون به دام افتد، تحمل بايدش

*(This poem is not translated to English, because Dr. Sadrizadeh often said, translations ruin the real feel and meaning of Hafez, even if they are done well).*

 May his memory be cherished, and may God bless his soul.
